# Navigating Primary Immune Thrombocytopenia During Pregnancy: Management Strategies and Considerations: A Comprehensive Review

**DOI:** 10.7759/cureus.67284

**Published:** 2024-08-20

**Authors:** Bhavana V Waghmare, Shubhada Jajoo

**Affiliations:** 1 Obstetrics and Gynaecology, Jawaharlal Nehru Medical College, Datta Meghe Institute of Higher Education and Research, Wardha, IND

**Keywords:** autoimmune disorder, neonatal thrombocytopenia, thrombocytopenia, management strategies, pregnancy, primary immune thrombocytopenia (itp)

## Abstract

Primary immune thrombocytopenia (ITP) is an autoimmune disorder characterized by a low platelet count due to the immune system's destruction of its platelets. During pregnancy, ITP poses significant challenges due to the need to balance maternal and fetal health. This comprehensive review aims to explore the pathophysiology, diagnostic approaches, and management strategies for ITP in pregnant women and discuss emerging treatments and future research directions. A thorough examination of current literature was conducted, including studies on the epidemiology, pathophysiology, diagnostic criteria, and treatment options for ITP in pregnancy. Relevant guidelines and expert consensus were also reviewed to provide a comprehensive understanding of best practices for managing this condition. The management of ITP in pregnancy requires a multidisciplinary approach and individualized treatment plans. First-line therapies include corticosteroids and intravenous immunoglobulin (IVIG), with second-line options such as thrombopoietin receptor agonists and immunosuppressive agents reserved for refractory cases. The choice of treatment depends on the severity of thrombocytopenia, the presence of bleeding symptoms, and gestational age. Special considerations include the risk of neonatal thrombocytopenia and the need for careful monitoring during labor and delivery. Emerging therapies and novel research offer promising advancements, though further studies are needed to validate their safety and efficacy. ITP in pregnancy is a complex condition that necessitates a careful balance between treating the mother and protecting the fetus. The management strategies must be tailored to each patient's needs, minimizing risks and optimizing outcomes. Continued research into the underlying mechanisms and treatment options will be crucial to improving care for pregnant women with ITP. This review provides a detailed synthesis of current knowledge and offers practical guidance for healthcare providers managing ITP during pregnancy.

## Introduction and background

Primary immune thrombocytopenia (ITP) is an autoimmune disorder characterized by a decrease in platelet count (thrombocytopenia) due to the body's immune system mistakenly targeting and destroying its platelets [1]. This immune-mediated destruction primarily involves autoantibodies directed against platelet surface antigens, notably glycoproteins IIb/IIIa and Ib/IX, leading to their recognition and phagocytosis by splenic macrophages. In some cases, reduced platelet production in the bone marrow can also play a role. The condition manifests with symptoms ranging from mild bruising and petechiae to more severe bleeding episodes, depending on the degree of thrombocytopenia [2]. ITP is a relatively rare disorder, with an estimated incidence of 2-5 per 100,000 adults annually. It can occur at any age but is most commonly diagnosed in young women, particularly during their reproductive years [3]. In the context of pregnancy, ITP accounts for about 5% of all cases of thrombocytopenia. The incidence in pregnant women is approximately 1-2 per 1,000 pregnancies, making it a significant concern for obstetricians and healthcare providers involved in maternal-fetal medicine [4].

The management of ITP during pregnancy poses unique challenges and is associated with specific risks for both the mother and the fetus. Pregnant women with ITP may face an increased risk of bleeding complications, particularly during labor and delivery, which can necessitate interventions such as platelet transfusions [5]. The condition also requires careful monitoring and management to balance the risks of treatment-related side effects against the benefits of maintaining a safe platelet count. For the fetus, maternal ITP can lead to neonatal thrombocytopenia, as maternal autoantibodies can cross the placenta and target fetal platelets. This can result in bleeding complications at birth, including intracranial hemorrhage in severe cases [6]. Given these complexities, this review aims to provide a comprehensive overview of the current understanding and management strategies for ITP during pregnancy. The objectives include discussing pathophysiology, diagnostic approaches, and therapeutic options and exploring the latest research and emerging treatments. By synthesizing the most recent evidence and clinical practices, this review seeks to offer practical guidance for healthcare providers and improve outcomes for both mothers and their babies.

## Review

Pathophysiology of ITP in pregnancy

Primary immune thrombocytopenia (ITP) is primarily characterized by an autoimmune response that destroys platelets. In pregnant women, this pathophysiology involves several fundamental immunological mechanisms [7]. The condition is driven by the production of IgG anti-platelet autoantibodies, which specifically target platelet glycoproteins, such as glycoprotein IIb/IIIa. These antibodies bind to platelets, marking them for destruction, predominantly in the spleen, where they interact with macrophage Fcγ receptors. This process accelerates platelet clearance from the circulation [8]. Additionally, T-cell involvement plays a significant role in the pathogenesis of ITP. An abnormal response from T follicular helper cells can lead to the proliferation of autoreactive B cells that produce these autoantibodies [9]. This immune dysregulation not only contributes to the destruction of platelets but may also impair platelet production. Immune-mediated damage to megakaryocytes, the precursor cells responsible for platelet generation, can further complicate the condition. Low levels of thrombopoietin exacerbate this, a crucial growth factor for megakaryocyte development, which can reduce platelet counts [9]. Pregnancy significantly influences the course of ITP, presenting unique challenges for management. The clinical presentation of ITP in pregnant women can vary widely; some may experience exacerbations, while others may see remissions. This variability is particularly pronounced in women with a pre-existing diagnosis of ITP [10]. Physiological changes during pregnancy, such as increased plasma volume and hormonal fluctuations, can also affect platelet counts. While many women experience a benign drop in platelet levels, it is crucial to differentiate this from ITP to ensure appropriate management [11]. Moreover, maternal antibodies can cross the placenta, potentially leading to neonatal thrombocytopenia. Approximately 10-15% of infants born to mothers with ITP may present with low platelet counts. While this condition can be concerning, severe complications, such as intracranial hemorrhage, are relatively rare, especially with careful monitoring and management [12]. The implications of ITP during pregnancy are significant for both maternal and fetal health. For mothers, the risk of bleeding complications increases, mainly if platelet counts drop below 20,000/µL. However, with appropriate management, the incidence of severe maternal hemorrhage remains low. Healthcare providers must closely monitor platelet levels and develop a tailored management plan that addresses each patient's needs [4]. For the fetus, while neonatal thrombocytopenia is a concern, the overall risk of severe outcomes is minimal when the condition is effectively managed. The incidence of serious complications, such as intracranial hemorrhage in neonates, is low, particularly when maternal platelet counts are monitored and maintained at safe levels during delivery. In summary, the pathophysiology of ITP in pregnancy involves complex immunological mechanisms that significantly impact maternal and fetal health, necessitating a multidisciplinary approach to ensure optimal outcomes for both mother and child [13].

Diagnosis of ITP in pregnant women

Immune thrombocytopenia (ITP) can manifest at any stage of pregnancy, whether the condition was known before conception or developed during gestation. Pregnant women with ITP may experience more significant risks of bleeding compared to non-pregnant individuals, particularly when platelet counts drop below 20-30 x 10^9/L. It is important to note that platelet counts typically decrease modestly during normal pregnancy; thus, many women with ITP may experience further reductions in platelet levels throughout their pregnancy. This necessitates careful monitoring and management to ensure maternal and fetal safety [14]. Diagnosing ITP in pregnant women requires distinguishing it from other causes of thrombocytopenia, as ITP is primarily a diagnosis of exclusion. The likelihood of ITP increases with decreasing platelet counts, but no definitive cutoff universally applies. Notably, platelet antibody tests are unreliable for differentiating ITP from incidental thrombocytopenia, which can occur during pregnancy [15]. A thorough medical history is essential; a history of prior thrombocytopenia, autoimmune diseases, or severe thrombocytopenia (defined as counts below 50 x 10^9/L) raises suspicion for ITP. Additionally, significant thrombocytopenia occurring in the first trimester with a progressive decline in counts is more suggestive of ITP, while mild thrombocytopenia in the second or third trimester, particularly in the absence of hypertension or proteinuria, is likely to represent incidental thrombocytopenia [7]. The diagnostic process for ITP in pregnant women primarily involves blood tests. A complete blood count (CBC) is essential for assessing platelet levels. In contrast, a peripheral blood smear can help rule out pseudothrombocytopenia, where platelet counts appear low due to clumping. Coagulation studies are also crucial in evaluating disseminated intravascular coagulation (DIC), a severe condition that can occur in pregnancy. Additionally, liver function tests may be performed to rule out conditions such as preeclampsia or HELLP syndrome, which can present with similar symptoms [10]. In some cases, a bone marrow examination may be considered to exclude other causes of thrombocytopenia, although it is not routinely required for diagnosing ITP in pregnancy. Imaging studies, such as ultrasound or MRI of the abdomen, may be utilized to assess for splenomegaly, which can indicate an underlying hematological condition. Overall, a comprehensive approach that includes clinical evaluation, blood tests, and, when necessary, further diagnostic procedures is essential for accurately diagnosing ITP in pregnant women [16].

Management strategies for ITP during pregnancy

**Figure 1:**
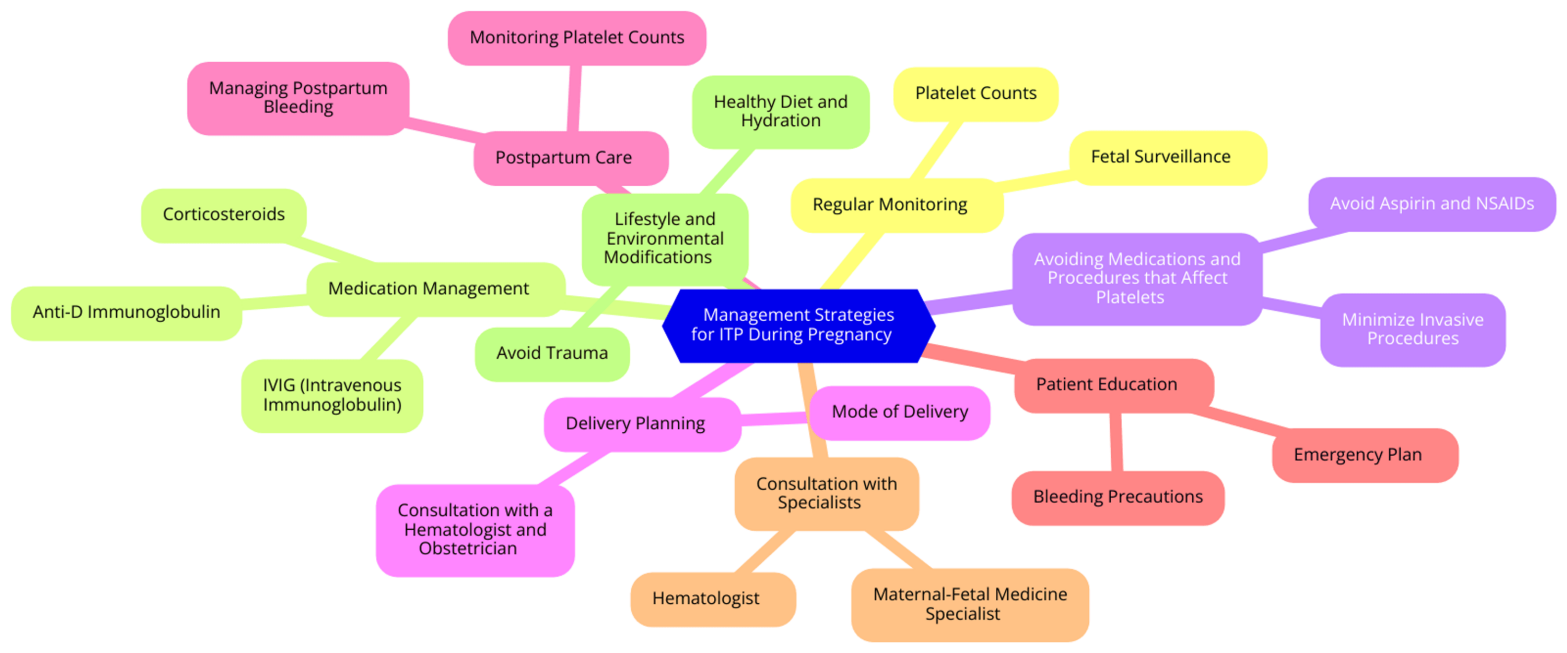
Management strategies for ITP during pregnancy Image Credit: Dr. Bhavana V. Waghmare

Managing primary immune thrombocytopenia (ITP) during pregnancy involves a collaborative approach among obstetricians, hematologists, and pediatricians. Close monitoring is essential, with regular assessments of platelet counts and maternal health. The goal is to maintain adequate platelet levels to prevent pregnancy, labor, and delivery complications. Platelet counts of 20-30 × 10^9/L are generally considered safe during most of the pregnancy, while a count of ≥50 × 10^9/L is preferred for delivery [4]. Corticosteroids, particularly prednisone, are the first-line treatment for ITP in pregnancy due to their efficacy and safety profile. They are typically administered at the lowest effective dose to minimize potential adverse effects on both the mother and the fetus. The initial dose usually ranges from 10-20 mg/day, with adjustments based on clinical response and platelet counts [17]. Intravenous immunoglobulin (IVIG) can rapidly increase platelet counts, especially in severe thrombocytopenia or before delivery. It is often combined with corticosteroids for a synergistic effect. IVIG is considered safe during pregnancy, although its effects on platelet counts are usually transient [18]. Immunosuppressive agents such as azathioprine may be considered when first-line treatments are ineffective. However, their use is limited due to potential risks to the fetus. Rituximab may be used in severe cases of ITP during pregnancy. Still, it carries perinatal and neonatal immunosuppression risks, necessitating careful newborn monitoring for infections and other complications [19]. Thrombopoietin receptor agonists (TPO-RAs) are generally not recommended during pregnancy due to limited safety data and potential risks to the fetus. They may be considered in late pregnancy if other treatments have failed, but this should be cautiously approached [20]. The severity of thrombocytopenia significantly influences treatment decisions. Patients with platelet counts <20 × 10^9/L or those experiencing bleeding typically require treatment, while those with higher counts may be monitored unless symptoms develop [10]. The timing of treatment is crucial, especially as delivery approaches. More aggressive treatment is often warranted in the third trimester to prepare for labor and minimize hemorrhagic risks. When selecting treatments, the potential risks to both mother and fetus must be weighed. Corticosteroids are generally considered safe, while other agents like rituximab and TPO-RAs require careful consideration due to their potential adverse effects [21]. Management strategies for ITP during pregnancy are shown in Figure 1.

Obstetric considerations

Managing primary immune thrombocytopenia (ITP) during pregnancy requires meticulous planning and coordination across various stages of care, including antenatal, intrapartum, and postpartum considerations. Each stage demands a tailored approach to ensure the health and safety of both the mother and the fetus [4]. Antenatal care for pregnant individuals with ITP focuses on regular monitoring and follow-up. Platelet counts should be assessed frequently, typically every four weeks during the first and second trimesters, with increased frequency as the pregnancy progresses, particularly in the third trimester. This consistent monitoring is crucial for timely intervention if platelet counts drop significantly. Additionally, patients should be educated about recognizing symptoms of bleeding or bruising, which may indicate worsening thrombocytopenia. Treatment plans should be individualized based on specific platelet counts, symptoms, and responses to therapy [4]. Multidisciplinary teams are vital in managing ITP during pregnancy. Collaboration among obstetricians, hematologists, and pediatricians ensures comprehensive care that prioritizes maternal and fetal health. This teamwork facilitates effective communication and shared decision-making, allowing for the development of a cohesive care plan. Furthermore, patient education is essential; healthcare providers should inform patients about ITP, potential complications, and the importance of adhering to follow-up appointments and treatment regimens [22].

Intrapartum management of ITP involves careful consideration of the mode of delivery. Vaginal delivery is generally preferred if platelet counts are above 30×10^9/L and there are no other contraindications. However, a cesarean section may be indicated if platelet counts are below 50×10^9/L or if additional risk factors, such as significant maternal bleeding or fetal distress, are present [10]. Anesthesia considerations are also crucial; neuraxial anesthesia, such as epidural or spinal anesthesia, may be considered if platelet counts are ≥70-80×10^9/L. In cases where platelet counts are too low for neuraxial anesthesia or if rapid intervention is required, general anesthesia may be necessary [23]. Management of hemorrhage during delivery is another critical aspect of intrapartum care. The delivery team should be prepared for potential hemorrhage, especially if the mother has low platelet counts. Establishing protocols for platelet transfusions and other blood products is essential to ensure prompt treatment for significant bleeding [24]. Postpartum care for mothers with ITP involves continued monitoring and treatment adjustments based on platelet counts. After delivery, platelet counts should be monitored closely, as they may fluctuate significantly. Many patients experience a rebound in platelet counts postpartum, but treatment may need to be adjusted based on the mother's clinical status and platelet levels. Corticosteroids or intravenous immunoglobulin (IVIG) may be continued to manage persistent thrombocytopenia [25]. Neonatal considerations are also important, as newborns of mothers with ITP are at risk for neonatal thrombocytopenia. It is essential to monitor the platelet counts of newborns within the first few days after birth. If thrombocytopenia is detected, management may include monitoring, IVIG, or platelet transfusions, depending on the condition's severity and the infant's clinical presentation [26].

Fetal and neonatal outcomes

Neonatal thrombocytopenia poses a significant risk for infants born to mothers with immune thrombocytopenia (ITP). Research shows that around 3.5% of neonates born to these mothers develop neonatal alloimmune thrombocytopenia (NAITP), characterized by a platelet count below 150×10^9/L at birth [27]. The risk of severe thrombocytopenia with a platelet count below 50×10^9/L can be as high as 37.5% in specific populations. This increased risk is often linked to maternal platelet counts, with lower counts at delivery correlating to a greater likelihood of neonatal thrombocytopenia. Therefore, monitoring maternal platelet levels during pregnancy is crucial to anticipate and manage potential neonatal complications [10]. Monitoring neonates born to mothers with ITP is critical, especially during the first week after birth, when platelet counts can decline significantly. Healthcare providers should regularly assess these infants' platelet levels to detect severe thrombocytopenia [28]. When thrombocytopenia is identified, immediate management may involve administering intravenous immunoglobulin (IVIG) or platelet transfusions, mainly if the platelet count is critically low or signs of bleeding are present. Close observation is essential to manage potential complications like intracranial hemorrhage (ICH), which can occur in neonates with severe thrombocytopenia. By maintaining vigilant monitoring and timely interventions, healthcare providers can significantly enhance outcomes for affected neonates [29]. Long-term outcomes for neonates born to mothers with ITP are generally favorable, though there remains an elevated risk for complications such as ICH and other bleeding disorders. Most affected neonates recover well from neonatal thrombocytopenia with appropriate management, and most do not experience long-term sequelae [30]. However, ongoing follow-up is recommended to monitor for potential developmental or health issues that may emerge. Early intervention can promptly address concerns, ensuring these infants achieve optimal health outcomes. Overall, while the potential for severe outcomes exists, with diligent monitoring and management, many neonates born to mothers with ITP can expect positive long-term health trajectories [31].

Special considerations and emerging therapies

Women diagnosed with immune thrombocytopenia (ITP) before pregnancy should receive thorough preconception counseling. This counseling should include a detailed review of the patient’s medical history, including specifics of their ITP, previous treatments, splenectomy history, obstetric history, and neonatal outcomes from prior pregnancies. Patients should be informed about potential complications during pregnancy and the necessity of regular platelet count monitoring. Approximately one-third of pregnant women with ITP may require treatment during their pregnancy. Despite the inherent risks, most women can proceed with pregnancy safely, particularly with appropriate management strategies [32]. Splenectomy, the surgical removal of the spleen, is a potential treatment option for women with refractory ITP. When considering splenectomy during pregnancy, it is generally advised to perform the procedure in the second trimester to minimize risks to both the mother and the developing fetus [33]. However, the decision to proceed with splenectomy should be made cautiously, weighing the potential benefits against the risks. Possible complications from the surgery include infections and bleeding, which can present significant challenges during pregnancy. Therefore, a multidisciplinary approach involving hematologists and obstetricians is crucial to ensure the safety and well-being of both mother and fetus [33]. Emerging therapies for managing ITP during pregnancy include immunosuppressants such as azathioprine and cyclosporine, which have demonstrated acceptable safety profiles in some cases. However, thrombopoietin receptor agonists, such as eltrombopag and romiplostim, are not routinely recommended due to insufficient safety data regarding their use during pregnancy. Research is ongoing to better understand the safety and efficacy of these treatments and to establish optimal management strategies for pregnant women with ITP [34]. For example, rituximab, a monoclonal antibody, may be considered in refractory cases but carries risks such as prolonged lymphopenia in newborns. Continued investigation into the long-term effects of corticosteroids and other treatments on maternal and fetal health is essential for improving ITP management during pregnancy. A careful, individualized approach is vital for optimizing mothers' and babies' outcomes [35].

Case studies and clinical scenarios

One illustrative case involved a pregnant woman with severe gestational thrombocytopenia [10]. A study reviewing 199 pregnancies with moderate to severe thrombocytopenia found that outcomes were generally favorable when the underlying cause was identified as gestational thrombocytopenia (GT) or immune thrombocytopenia (ITP). The management of this case included meticulous monitoring of platelet counts and prompt interventions when levels fell below critical thresholds. This case underscores the importance of an accurate diagnosis and the need for tailored management strategies that address the specific etiology of thrombocytopenia during pregnancy [36]. Another notable case involved the management of a pregnant patient with ITP. In this instance, treatment included administering prednisone at the lowest effective dose, complemented by intravenous immunoglobulin (IVIG) for rapid response when platelet counts were critically low. This case highlighted the necessity of a multidisciplinary approach, with close collaboration between obstetricians and hematologists, to ensure the safety and well-being of both mother and fetus throughout the pregnancy [18].

A series of cases also documented the incidence of gestational thrombocytopenia, the most common form of thrombocytopenia during pregnancy. These cases emphasized distinguishing gestational thrombocytopenia from other underlying causes, such as ITP or systemic disorders. Accurate differentiation is crucial to avoid unnecessary interventions and to ensure appropriate monitoring of both maternal and neonatal health [10]. From these case studies, several key lessons and best practices emerge. First, early diagnosis and monitoring are vital. Routine screening for thrombocytopenia during pregnancy enables the early identification of potential issues. Regular monitoring of platelet counts is essential, especially for patients with counts below 80,000/mm³, which necessitates further evaluation to rule out ITP or other causes [37].

Another important lesson is the value of multidisciplinary collaboration. Managing ITP and other thrombocytopenic disorders during pregnancy requires a coordinated effort among obstetricians, hematologists, and pediatricians. This teamwork ensures comprehensive care that addresses maternal health and fetal outcomes, leading to more effective management strategies [4]. Individualized treatment plans are also crucial. Each patient's treatment strategy should be tailored to her specific condition, considering the severity of thrombocytopenia and associated symptoms. While prednisone is commonly used as the first-line treatment, alternatives such as IVIG should be available for cases requiring rapid intervention [15]. Patient education and support are essential in managing thrombocytopenia during pregnancy. Educating patients about their condition, including potential risks to the fetus, helps alleviate anxiety and improve adherence to monitoring protocols. Emotional support and clear communication are vital for a positive patient experience [10]. Finally, postpartum monitoring is critical. Continuous assessment of both mother and neonate after delivery is necessary, as platelet levels can fluctuate significantly in the early postpartum period. Establishing protocols for neonatal platelet assessment can aid in the early detection of any cases of fetal thrombocytopenia, ensuring timely intervention and care [38].

Ethical and psychosocial considerations

Managing primary immune thrombocytopenia (ITP) during pregnancy involves complex ethical considerations concerning patient autonomy and informed decision-making. Pregnant patients with ITP must be thoroughly informed about their condition, treatment options, and the potential risks to both them and their fetus. This includes detailed discussions about corticosteroids and intravenous immunoglobulin (IVIG) treatments, which may have side effects and varying efficacy during pregnancy. Ensuring patients fully understand these factors is crucial for obtaining informed consent [39]. A collaborative approach between obstetricians and hematologists is essential in this context. This partnership ensures the patient's values and preferences are respected while balancing clinical recommendations. Patients should be encouraged to actively participate in discussions about their care plan, including decisions regarding the timing of interventions and delivery methods. Ethical dilemmas may arise when patients' cultural or religious beliefs conflict with medical recommendations. Healthcare providers must navigate these situations sensitively, respecting patients' beliefs while delivering appropriate medical care [40]. The psychological impact of ITP on pregnant patients can be profound, affecting their mental health and overall well-being. The diagnosis of ITP can lead to heightened anxiety about the health of both the mother and the fetus. Concerns about potential complications, such as severe bleeding or neonatal thrombocytopenia, can exacerbate stress. Many patients worry about their ability to manage the condition effectively during pregnancy, which can contribute to a sense of helplessness [41]. Providing psychological support is essential to addressing these concerns. Healthcare providers should facilitate access to counseling services and support groups for pregnant women with ITP. Such support can help mitigate feelings of isolation and anxiety by allowing patients to share their experiences and coping strategies. Additionally, the psychological effects often continue beyond delivery; patients may experience ongoing anxiety related to postpartum ITP management and the potential for neonatal complications. Continuous mental health support is vital during this transition period to ensure that mothers feel prepared to manage their health and that of their newborns [42].

## Conclusions

In conclusion, managing primary immune thrombocytopenia (ITP) during pregnancy presents a complex interplay of risks and challenges that necessitate a careful, individualized approach. The autoimmune nature of ITP, combined with the physiological changes of pregnancy, requires healthcare providers to balance maternal safety with fetal well-being. Effective management involves a deep understanding of the disease's pathophysiology and an accurate diagnosis, followed by a tailored therapeutic strategy. Treatment options, ranging from corticosteroids and intravenous immunoglobulin to thrombopoietin receptor agonists and immunosuppressive agents, must be selected based on the severity of thrombocytopenia and the stage of pregnancy. The involvement of a multidisciplinary team, including obstetricians, hematologists, and pediatricians, is crucial for optimizing both maternal and neonatal outcomes. As research progresses and new therapies emerge, there is a growing potential to improve treatment efficacy and safety. However, it remains essential to maintain a patient-centered approach, ensuring that management decisions are guided by the best available evidence and adapted to each patient's specific circumstances. This comprehensive review highlights the current state of knowledge and practice, emphasizing the need for continued research to enhance our understanding and treatment of ITP during pregnancy.
